# Neutrophil-to-Lymphocyte Ratio and Platelet-to-Lymphocyte Ratio in Blood to Distinguish Lung Cancer Patients from Healthy Subjects

**DOI:** 10.1155/2020/8844698

**Published:** 2020-10-16

**Authors:** Xuming Zhu, Huizhu Song, Yan Chen, Feifei Han, Qiong Wang, Yubao Cui

**Affiliations:** ^1^Department of Clinical Laboratory, Wuxi People's Hospital Affiliated with Nanjing Medical University, Wuxi, 214023 Jiangsu Province, China; ^2^Department of Pharmacy, Wuxi People's Hospital Affiliated with Nanjing Medical University, Wuxi, 214023 Jiangsu Province, China

## Abstract

**Objective:**

Inflammation-driven markers play a crucial role in tumorigenesis and tumor progression. The neutrophil-to-lymphocyte ratio (NLR) and platelet-to-lymphocyte ratio (PLR) in blood are systemic inflammatory response markers. Some reports have showed that NLR and PLR are related to a poor prognosis in patients with lung cancer. However, little studies have reported whether NLR and PLR can be diagnostic markers for lung cancer. The aim of the current study is to investigate the roles of NLR and PLR in diagnosing lung cancer.

**Methods:**

This study analyzed data from lung cancer patients and healthy individuals in Wuxi People's Hospital Affiliated with Nanjing Medical University. The Mann–Whitney *U* test was performed to compare differences between the lung cancer group and the control group. Based on white blood cell (WBC) counts, both lung cancer patients and healthy individuals were divided into the low-level group, moderate-level group, and high-level group. The Kruskal-Wallis test was applied to compare differences of NLR and PLR among those groups with different WBC counts. Spearman correlation analysis was used to assess correlations. Receiver operating characteristic (ROC) curves were performed to determine diagnostic accuracy.

**Results:**

210 patients diagnosed with lung cancer and 261 healthy subjects were enrolled in this study. Levels of NLR and PLR increased in the lung cancer group compared with the control group (*P* < 0.001). For the lung cancer group, NLR levels could rise with the increasing of WBC levels (*P* < 0.001) while PLR levels had no significant variation with the increasing of WBC levels (*P* = 0.206). For the control group, NLR levels could rise with the increasing of WBC levels (*P* < 0.001) while PLR levels would decline with the increasing of WBC levels (*P* < 0.001). In the lung cancer group, both NLR and PLR had no significant correlations with aspartate transaminase, urea, and glucose. The area under the curve (AUC) with 95% confidence interval (95% CI) of NLR and PLR to distinguish lung cancer patients from healthy subjects was, respectively, 0.684 (0.634-0.735) and 0.623 (0.571-0.674). When NLR and PLR were combined, AUC (95% CI) increased to 0.691 (0.642-0.740).

**Conclusions:**

NLR and PLR alone have moderate ability to distinguish lung cancer patients from healthy subjects. Furthermore, combination forms of NLR and PLR can improve diagnostic ability.

## 1. Introduction

According to Global Cancer Statistics 2018, lung cancer is a leading cause of newly diagnosed cancer and deaths across 20 regions of the world [1]. In 2019, the American Cancer Society estimated in the United States that there were 116,440 and 111,710 new lung cancer cases with 24% and 23% of new deaths per year for men and women, respectively [2]. Lung cancer represents a major worldwide disease burden and will continue to be a major health problem through the first half of this century [3].

Inflammation is an important component of the tumor microenvironment, and selected chronic inflammatory conditions increase the risk of developing cancer [4]. Inflammation-driven markers play a crucial role in tumorigenesis and tumor progression [5]. For example, the systemic inflammation score, a systemic inflammatory marker based on the lymphocyte-to-monocyte ratio and serum albumin level, might serve as an independent biomarker for predicting adverse events and prognosis in locally advanced rectal cancer [6].

The neutrophil-to-lymphocyte ratio (NLR) and platelet-to-lymphocyte ratio (PLR) are well-known systemic inflammatory response markers. Some reports have showed that NLR and PLR are related to poor prognosis in patients with lung cancer. A meta-analysis demonstrated that high pretreatment NLR was closely related to poorer progression-free survival and overall survival in patients with small-cell lung cancer [7]. A retrospective descriptive study showed that high PLR was significantly associated with poor overall survival in patients with stage IV non-small-cell lung cancer [8]. As for the diagnostic ability, both NLR and PLR have moderate abilities to detect ovarian cancer patients from healthy controls [9]. However, little studies have reported whether NLR and PLR in blood can be diagnostic biomarkers for lung cancer. The aim of the current study is to investigate the roles of NLR and PLR, either alone or combined, in diagnosing lung cancer.

## 2. Materials and Methods

### 2.1. Study Population

Data from participants at Wuxi People's Hospital Affiliated with Nanjing Medical University between August 2019 and November 2019 were retrospectively analyzed. Patients diagnosed with lung cancer according to medical records were enrolled into the lung cancer group. Patients with diabetes mellitus, acute inflammation, cirrhosis, coronary artery disease, kidney disease, and other malignant tumors were excluded. Healthy subjects were regarded as the control group. This study was approved by the ethics committee of Wuxi People's Hospital Affiliated with Nanjing Medical University. Patient consent was waived due to the retrospective nature of this paper. This study was conducted following the Declaration of Helsinki.

### 2.2. Laboratory Assays

After fasting for at least 8 hours, venous blood (5 mL) was collected from each participant in the morning and placed in EDTA-K2 vacuum anticoagulation tubes and drying tubes. Whole blood samples of EDTA-K2 tubes were analyzed in a Sysmex XE-5000 Automatic Hematology Analyzer (Sysmex Corp., Kobe, Japan) to determine whole blood routine parameters. The total number of white blood cell (WBC), hemoglobin (HGB), red blood cell distribution width (RDW), and platelet distribution width (PDW) were obtained directly from an analyzer. Additional levels of the neutrophil-to-lymphocyte ratio (NLR) and platelet-to-lymphocyte ratio (PLR) were obtained indirectly through calculation. Blood samples of drying tubes were allowed to clot at room temperature for 60 minutes, followed by centrifugation at 3000 × g for 3 min to obtain serum. Serum samples were analyzed within 2 hours by a Beckman AU5800 Automatic Analyzer (Beckman Coulter Inc., CA, USA) to detect biochemical markers including aspartate transaminase (AST), urea (UR), and glucose (GLU).

### 2.3. Statistical Analysis

All continuous variables were not complied with normal distributions using the Kolmogorov-Smirnova test, and continuous variables were presented as the median (interquartile range). The categorical variable was expressed as the number (percentage). The Mann–Whitney *U* test and chi-squared test were, respectively, performed to compare statistical differences in continuous variables and categorical variable between the lung cancer group and the control group. The Kruskal-Wallis test was applied to compare statistical differences of NLR and PLR among three groups with different WBC counts. Spearman correlation analysis was used to assess correlations between NLR, PLR, and biochemical markers. The receiver operating characteristic (ROC) curve was constructed, and the area under the curve (AUC) with 95% confidence interval (95% CI) was calculated to determine diagnostic accuracy. Values of *P* < 0.05 were considered statistically significant. All statistical analyses were performed by SPSS version 20.0 (SPSS Inc., Chicago, USA).

## 3. Results

### 3.1. Differences between the Lung Cancer Group and the Control Group

A total of 210 lung cancer patients and 261 healthy subjects were enrolled in this paper, and their characteristics are presented in Table 1. Age (*P* = 0.539) and gender (*P* = 0.366) showed no significant differences between the lung cancer group and the control group, proving that they were age-matched and gender-matched. HGB, RDW, and PDW were significantly lower in the lung cancer group compared to the control group (*P* < 0.001), while WBC was significantly higher in the lung cancer group (*P* = 0.001). NLR and PLR, two calculated parameters, were significantly higher in the lung cancer group compared to the control group (*P* < 0.001). Levels of biochemical markers such as AST (*P* = 0.003) and GLU (*P* < 0.001) were lower in the lung cancer group compared to the control group, while UR was higher in the lung cancer group (*P* = 0.024).

### 3.2. Comparison of NLR and PLR among Different WBC Groups

According to WBC levels, both the lung cancer group and the control group were divided into the low-level group (WBC < 4.9 × 10^9^/L), moderate-level group (4.9 × 10^9^/L ≤ WBC ≤ 5.8 × 10^9^/L), and high-level group (WBC > 5.8 × 10^9^/L). Levels of NLR and PLR were compared among these groups with different WBC levels (Table 2). For the lung cancer group, NLR levels could rise with the increasing of WBC levels (*P* < 0.001) while PLR levels had no significant variation with the increasing of WBC levels (*P* = 0.206). For the control group, NLR levels could rise with the increasing of WBC levels (*P* < 0.001) while PLR levels would decline with the increasing of WBC levels (*P* < 0.001).

### 3.3. Correlation Analysis

Results of correlation analysis between NLR, PLR, and biochemical markers are shown in Table 3. In the lung cancer group, both NLR and PLR had no significant correlations with all biochemical markers. In the control group, NLR had statistically significant negative weak correlations with AST (*r* = −0.134, *P* = 0.03) and UR (*r* = −0.146, *P* = 0.018). Additionally, PLR had statistically significant negative weak correlation with AST (*r* = −0.208, *P* = 0.001), UR (*r* = −0.186, *P* = 0.003), and GLU (*r* = −0.179, *P* = 0.004).

### 3.4. Diagnostic Accuracy of NLR and PLR

**Figure 1:**
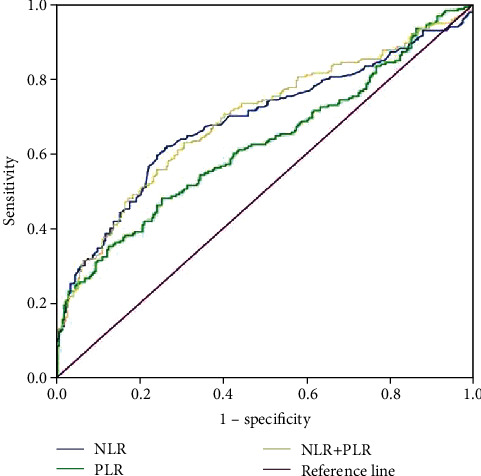
ROC curve analysis of the NLR and PLR alone or combination of NLR and PLR.

Diagnostic values of NLR and PLR to distinguish lung cancer patients from healthy subjects are presented in Table 4 and Figure 1. With an optimal cutoff point of 2.14, SEN and SPE of NLR were, respectively, 0.619 and 0.736. With an optimal cutoff point of 149.95, SEN and SPE of PLR were, respectively, 0.481 and 0.747. NLR and PLR had, respectively, AUC (95% CI) of 0.684 (0.634-0.735) and 0.623 (0.571-0.674). When NLR and PLR were combined, AUC (95% CI) increased to 0.691 (0.642-0.740).

## 4. Discussion

Tumor-infiltrating inflammatory cells mediate processes associated with progression, invasion, and metastasis [10]. Immunoregulatory cytokines secreted in a proinflammatory environment also contribute to tumor growth and metastases [11]. With the assistance of cytokines, cancer cells might facilitate recruitment of tumor-associated neutrophils, which further help the tumor metastasis. Instead, lymphocytes are faithful anticancer defenders, and high lymphocyte counts have been proved as a favorable factor in terms of survival in a good way in many human cancers [12]. NLR in peripheral blood is being increasingly studied as a systemic inflammatory marker, particularly considering its rapid, widely available, and relatively inexpensive assessment through routine blood count analysis [13]. Besides lung cancer, NLR has also been proved to be associated with prognosis of small renal cell carcinoma and breast cancer [14, 15]. The current study showed that NLR was higher in lung cancer patients compared to healthy subjects, confirming the role of NLR in the progression of lung cancer. Additionally, NLR levels could rise with the increasing of WBC levels in both lung cancer groups and control group, showing that WBC might have the same clinical significance with NLR.

Platelet and lymphocyte counts are basic hematological examinations that are very easy, cheap, and fast to apply [16]. Tumor cells can secrete platelet agonists to induce platelet aggregation, which results in thrombocytosis with playing a role in cancer genesis and development [17]. Additional, lymphocytes play a role by being anticancer defenders [18]. Therefore, PLR, the combination of these two parameters, may reflect a balance between tumor development and tumor suppression. Several reports have shown that PLR is an inflammatory marker used as a prognostic factor in lung cancer, hepatocellular carcinoma, and gastric cancer [8, 18, 19]. The current study showed that PLR was higher in lung cancer patients compared to healthy subjects, confirming the role of platelet activity and lymphocyte in the progression of lung cancer. Furthermore, in the lung cancer group, PLR levels had no significant variation with the increasing of WBC levels while in the control group, PLR levels would decline with the increasing of WBC levels, showing that PLR might be contrary to WBC in clinical significance.

AST, usually accompanying alanine transaminase, is a liver injury marker. UR and GLU are markers reflecting the status of metabolism in the body; monitoring their levels in blood is important for patients with diabetes or kidney diseases [20]. Correlation analysis in the current study demonstrated that in lung cancer patients, NLR and PLR were not correlated with AST, UR, and GLU, showing that NLR and PLR might not exert significant effect on the process of metabolism in lung cancer patients.

This study showed that NLR and PLR alone had moderate ability to distinguish lung cancer from healthy subjects. Furthermore, combined forms of NLR and PLR can increase their diagnostic value, presenting its capability to be a clinically accessible biomarker.

There are some limitations in the current paper. First, patients with lung cancer were not classified into different groups according to cancer stage, ignoring their different effects on conclusions. Second, therapy measures involving surgery, steroids therapy, chemotherapy [21], and radiotherapy were not evaluated, which could influence levels of blood routine parameters or biochemical markers. Third, the sample size in this paper was relatively small, preventing us from drawing firm conclusions. Fourth, some basic information of participants such as race and BMI was not enrolled, ignoring their role on inflammatory markers of the current paper.

Overall, NLR and PLR alone have moderate ability to distinguish lung cancer patients from healthy subjects. Furthermore, combination forms of NLR and PLR can improve diagnostic ability.

## Figures and Tables

**Table 1:** Characteristics of the study participants.

	Lung cancer group (*n* = 210)	Control group (*n* = 261)	*U* value	*P* value
Male (%)^∗^	118 (56.2)	154 (59.0)	0.377^∗∗^	0.539
Age (years)	65 (58-70)	61 (48-75)	26077.5	0.366
WBC (×10^9^/L)	5.9 (4.7-7.1)	5.4 (4.7-6.2)	22420.0	0.001
HGB (g/L)	126 (114-136)	143 (134-154)	11811.0	<0.001
RDW (%)	13.0 (12.3-14.1)	13.4 (13.0-13.9)	22159.0	<0.001
PDW (fL)	12.1 (10.5-13.9)	16.7 (16.4-17.1)	4478.0	<0.001
NLR	2.43 (1.67-3.48)	1.77 (1.39-2.20)	17290.0	<0.001
PLR	143.52 (97.46-207.97)	115.70 (93.50-151.27)	20680.0	<0.001
AST (U/L)	22 (18-28)	23 (20-28)	22979.5	0.003
UR (mmol/L)	5.7 (4.4-6.9)	5.2 (4.2-6.3)	24085.5	0.024
GLU (mmol/L)	5.39 (4.89-5.94)	5.59 (5.25-6.31)	21739.5	<0.001

^∗^Categorical variable; ^∗∗^chi-squared test value. Abbreviation: AST: aspartate transaminase; UR: urea; GLU: glucose; WBC: white blood cell; HGB: hemoglobin; RDW: red blood cell distribution width; PDW: platelet distribution width; NLR: neutrophil-to-lymphocyte ratio; PLR: platelet-to-lymphocyte ratio.

**Table 2:** Comparison of NLR and PLR among 3 different WBC groups.

		WBC < 4.9 (×10^9^/L)	4.9 ≤ WBC ≤ 5.8 (×10^9^/L)	WBC > 5.8 (×10^9^/L)	*H* value	*P* value
Lung cancer group	*n*	57	42	111		
	NLR	1.77 (1.25-2.61)	2.35 (1.53-3.27)	2.76 (2.14-4.15)	28.29	<0.001
	PLR	152.70 (107.54-206.93)	160.08 (99.7-220.52)	136.65 (93.32-187.28)	3.16	0.206
Control group	*n*	78	95	88		
	NLR	1.53 (1.21-2.01)	1.84 (1.48-2.11)	1.91 (1.44-2.52)	11.53	<0.001
	PLR	130.16 (107.17-161.77)	116.81 (97.28-157.23)	99.2 (77.67-130.35)	24.55	<0.001

**Table 3:** Results of correlation analysis.

	Lung cancer group (*n* = 210)		Control group (*n* = 261)	
	*r*	*P* value	*r*	*P* value
NLR and AST	-0.097	0.16	-0.134	0.03
NLR and UR	-0.062	0.368	-0.146	0.018
NLR and GLU	0.124	0.074	0.075	0.224
PLR and AST	-0.049	0.482	-0.208	0.001
PLR and UR	-0.088	0.201	-0.186	0.003
PLR and GLU	0.07	0.315	-0.179	0.004

**Table 4:** Evaluation of diagnostic value.

Variables	Cutoff point	Sensitivity	Specificity	AUC (95% CI)
NLR	2.14	0.619	0.736	0.684 (0.634-0.735)
PLR	149.95	0.481	0.747	0.623 (0.571-0.674)
NLR+PLR		0.629	0.693	0.691 (0.642-0.740)

## Data Availability

This is a retrospective paper. The data used to support the findings of this study are available from the corresponding author upon request.
